# Risks of Intra-Pleural Injections

**Published:** 1882-06

**Authors:** 


### Risks of Intra-Pleural Injections.

A few years ago we heard far more frequently of fatal accidents occurring during the operation of washing out an empyema than we have of late; but we are reminded of these risks in a note from Prof. Billroth's clinic in the *Allegemeine Wiener Med. Zeitung* for December 20th. The writer says that Professor Billroth has become convinced of the inutility of injections for the purpose of washing out the empyemic cavity, except in the case of blood-clots and decomposing secretion; and in the latter case it suffices to perform a single but thorough injection. Thus in one case of a shot-wound in the left thorax, leading to putrid empyema, Professor Billroth made a counter-opening, and for four days allowed thymol to flow through. In ordinary empyema the chances are favorable when the operation is done at the right time, for the longer pus remains in the thorax the longer the lung keeps atelectatic, and thus does not approach the wall of the thorax. A rib is resected, a drainage-tube introduced, and pus allowed free escape—a method of treatment much like that practiced by Hippocrates, who bored through the rib and introduced a short smooth metal tube into the opening. To diminish pus formation a rod of iodoform can be placed in the pus cavity. Injections of cold disinfecting fluids often lead to ill consequences. Profesor Billroth relates one—a female, twenty years old, with empyema, who was treated by means of injections. One day, when a cure was nearly accomplished, she became unconscious during the injection, and could not be restored. Dr. Wölfler also had an older patient who became unconscious during the injection, but who recovered. Billroth explains these remarkable phenomena, that a shock is received by the organism, excited through the peripheral nerves by means of cold water, and under ever so slight conditions, it may be the cause of death; just as a mere blow on the testicle or stomach region can be fatal. Therefore it is important to employ injections, when they appear necessary, of warm fluid.—*Lancet, Jan. 7, 1882.*

